# Nanoparticles without and with protein corona: van der Waals and hydration interaction

**DOI:** 10.1007/s10867-019-09530-8

**Published:** 2019-08-20

**Authors:** Vladimir P. Zhdanov

**Affiliations:** 1 Section of Biological Physics, Department of Physics, Chalmers University of Technology, Gothenburg, Sweden; 2 Boreskov Institute of Catalysis, Russian Academy of Sciences, Novosibirsk, Russia

**Keywords:** Nanoscience, Nanoparticles, Intermolecular forces, Aggregation

## Abstract

The van der Waals (vdW) interaction between nanoparticles (NPs) in general, and especially between metal NPs, may be appreciable, and may result in nanoparticle aggregation. In biofluids, NPs become rapidly surrounded by a protein corona (PC). Here, the vdW and hydration interaction of NPs with and without PC are compared in detail. The focus is on two widely used types of NPs fabricated of SiO_2_ and Au and possessing weak and strong vdW interactions, respectively. For SiO_2_, the presence of PC increases the vdW interaction, but it remains relatively weak and insufficient for aggregation. For Au, the presence of PC decreases the vdW interaction, and in the case of small NPs (≤ 40 nm in diameter) it may become insufficient for aggregation as well while the larger NPs can aggregate.

## Introduction

**Fig. 1:**
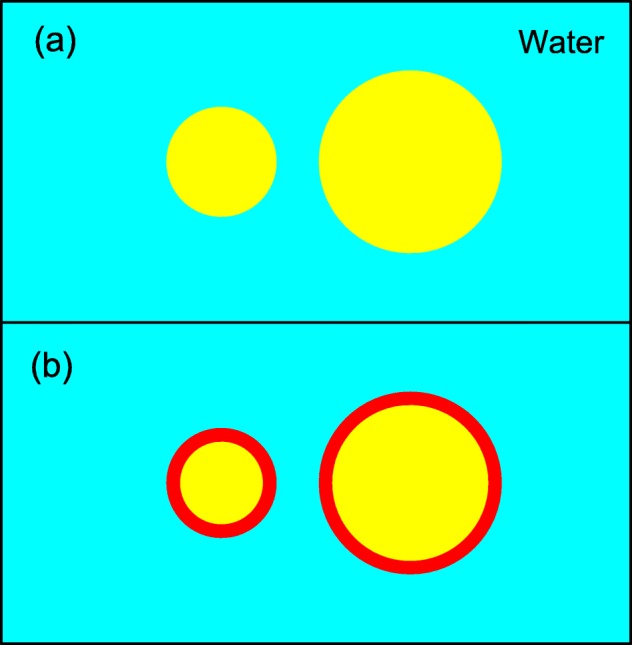
Schematic of nanoparticles **a** without and **b** with a protein corona. In the calculations presented below, the radius of nanoparticles is considered to be in the range from 20 to 80 nm, and the corona thickness is set to 5 nm

NPs have the potential for various biological and medical applications, including targeted drug delivery, hyperthermia therapy, and contrast imaging, and simultaneously may induce deterioration of some of the organism functions. For these reasons, the behavior of NPs in biofluids is now a subject of numerous experimental and theoretical studies (for seminal works and recent reviews, see Refs. [1, 2] and [3–7], respectively). In this context, it is of interest that the vdW attraction between NPs may result in their aggregation (for the kinetic models of aggregation, see Refs. [8–12]). In biofluids, NPs are usually surrounded by PC (Fig. 1; reviewed in [1–7]; for the corresponding mean-field kinetic models and typical molecular dynamics simulations, see Refs. [13–17] and [18–21], respectively). The presence of PC influences the interaction between NPs and may reduce the driving force for aggregation [7, 22]. Herein, I clarify this effect by scrutinizing and comparing the vdW and hydration interactions between NPs with and without PC.

Phenomenologically, the interaction of bare or PC-possessing NPs can be described by dividing the system into the vdW, hydration, and double-layer electrostatic parts, *U*_vdW_, *U*_h_, and *U*_dl_. The latter two forces operate on the length scale of $\simeq \!\!1$ nm, while the range of the former forces depends on the NP size and is appreciably larger. In general, all these forces should be taken into account. Bearing in mind the physiological in vivo conditions, one can, however, notice that the double-layer potential of proteins is usually rather low and not sufficient for aggregation. One of the indicators confirming this fact is, e.g., that the plasma proteins typically do not aggregate [23]. Another indicator is that various methods of calculation of the protein-protein interaction show that its scale is typically of several *k*_B_*T*, except for a small fraction of configuration space where it is up to tens of *k*_B_*T* [24]. Under the same conditions, the charge density and double-layer potential of bare NPs, fabricated, e.g., of Au or SiO_2_, are rather low as well [9, 25, 26] (on SiO_2_, e.g., the charge density is about − 0.1 C/m^2^ or 0.6 e/nm^2^ [25]). This means that *U*_dl_ is smaller than *U*_vdW_ and *U*_h_. Focusing on such situations, I neglect *U*_dl_. This approximation can be made at least outside the double layer (the length scale of which is roughly 1 nm). If needed, *U*_dl_ can be included into the analysis (one can find various expressions for this potential in the literature). From the perspective of aggregation induced by the vdW interaction, the role of *U*_h_ is typically not crucial (see below), and the role of *U*_dl_ is not crucial either. For this reason, the inclusion of *U*_dl_ is not expected to change my main conclusions.

To calculate *U*_vdW_, I use the conventional additive Hamaker approximation [27, 28]. In complex systems in general, the vdW interaction is well-known to be often nonadditive [29]. In the situations treated herein, the corresponding corrections are, however, not crucial because either one of its counterparts dominates or the vdW properties of some of the counterparts are similar (for more specific arguments, see below). To describe *U*_h_, I employ the empirical potential in combination with the Derjaguin approximation [27, 30]. Bare and PC-possessing NPs are considered to be spherical (Fig. 1). The corresponding equations used below (Section 2) are general. Illustrating their application (Sections 3 and 4), I describe SiO_2_ and Au NPs with weak and strong vdW interactions, respectively. The NPs of these types are widely employed in experiments, and accordingly the results presented below are instructive from this perspective.

## General equations

In my treatment, as already noticed in the Introduction, the interaction between two bare or PC-possessing NPs is represented as

1 $$ U = U_{\text{vdW}} + U_{\mathrm{h}}. $$

For bare NPs of radii *R*_1_ and *R*_2_ (Fig. 1a), one has [27, 28]

2 $$ U_{\text{vdW}}= - A_{\text{NP1-NP2}} \varphi (R_{1},R_{2},d) / 6 , $$

where *A*_NP1-NP2_ is the Hamaker constant, *d* is the minimal NP-NP distance, and

3 $$ \begin{array}{@{}rcl@{}} \varphi (R_{1},R_{2},d) &\equiv& \frac{2R_{1} R_{2}}{2(R_{1}+R_{2})d+d^{2}}\hspace{15 mm}\\ &&+ \frac{2R_{1} R_{2}}{4R_{1}R_{2}+2(R_{1}+R_{2})d+d^{2}} \\ &&+ \ln \left[ \frac{2(R_{1}+R_{2})d+d^{2}}{4R_{1}R_{2}+2(R_{1}+R_{2})d+d^{2}} \right] . \end{array} $$

The corresponding hydration energy is given by [27, 30]

4 $$ U_{\mathrm{h}}=\frac{2\pi B R_{1} R_{2} }{\alpha (R_{1}+R_{2})}\exp (- \alpha d), $$

where *α* and *B* are parameters determined via the energy of the interaction (per unit area) of the flat interfaces, $U_{h}=B\exp (- \alpha d)$.

For NPs surrounded by PC of thickness *h*_1_ and *h*_2_ (Fig. 1b), the vdW interaction can be represented as a sum of four terms corresponding to the core-core, core-shell, core-shell, and shell-shell parts,

5 $$ U_{\text{vdW}}= V_{\text{NP1-NP2}}+V_{\text{NP1-PC2}}+V_{\text{NP2-PC1}}+V_{\text{PC1-PC2}}, $$

and each term can be expressed via the function *φ*(*X*,*Y*,*Z*) defined by (3) (see, e.g., the prescriptions in [27, 28]),

6 $$ V_{\text{NP1-NP2}} = -A_{\text{NP1-NP2}}  \varphi (R_{1},R_{2},d+h_{1}+h_{2}) / 6, $$

7 $$ \begin{array}{@{}rcl@{}} V_{\text{NP1-PC2}} &=& -A_{\text{NP1-PC2}} [ \varphi (R_{1},R_{2}+h_{2},d+h_{1})\\ &&- \varphi (R_{1},R_{2},d+h_{1}+h_{2})]/ 6, \end{array} $$

8 $$ \begin{array}{@{}rcl@{}} V_{\text{NP2-PC1}} &=& -A_{\text{NP2-PC1}} [ \varphi (R_{1}+h_{1},R_{2},d+h_{2})\\ &&- \varphi (R_{1},R_{2},d+h_{1}+h_{2})]/ 6, \end{array} $$

9 $$ \begin{array}{@{}rcl@{}} V_{\text{PC1-PC2}} &=& -A_{\text{PC1-PC2}} [\varphi (R_{1}+h_{1},R_{2}+h_{2},d)\\ &&- \varphi (R_{1},R_{2}+h_{2},d+h_{1})\\ &&- \varphi (R_{1}+h_{1},R_{2},d+h_{2})\\ &&+ \varphi (R_{1},R_{2},d+h_{1}+h_{2})]/ 6, \end{array} $$

where *A*_NP1-NP2_, *A*_NP1-PC2_, *A*_NP2-PC1_, and *A*_NP2-PC1_ are the Hamaker constants.

The hydration energy can in the case under consideration be obtained by replacing *R*_1_ and *R*_2_ in (4) by *R*_1_ + *h*_1_ and *R*_2_ + *h*_2_,

10 $$ U_{\mathrm{h}}=\frac{2\pi B (R_{1}+h_{1}) (R_{2}+h_{2})}{\alpha (R_{1}+R_{2}+h_{1}+h_{2})} \exp (- \alpha d). $$

## Parameters

To calculate the vdW interaction, one needs the Hamaker constants. These constants characterize the interaction of two materials of interest and a medium between them. In my calculations, I use the constants obtained earlier experimentally or theoretically. As a rule, accurate determination of these constants is difficult. On the length scale of interest, strictly speaking, the Hamaker constants depend on the NP size (see, e.g., [31, 32]), but this effect is relatively weak (compared with the typical accuracy of the determination of the values of the Hamaker constants) and is below not taken into account. Another complicating factor is that in the situations with PC, the medium between NPs contains two phases, water and protein. The Hamaker constants for such situations are lacking. The screening properties of protein are, however, close to those of water (see, e.g., Refs. [33, 34] and [29], respectively). For this reason, I use the Hamaker constants for the interaction across water in all the cases. This approximation is rather accurate for metals (e.g., for Au) with appreciable polarizability and less accurate for SiO_2_ with smaller polarizability. In the latter case, the contribution of SiO_2_ to the vdW interaction of SiO_2_ NPs in the presence of PC is in any case rather low, and accordingly the approximation employed does not influence the results and conclusions.

For the Au-Au vdW interaction, the experimental and theoretical studies indicate that the Hamaker constant is in the range from 15 to 40 × 10^− 20^ J [32, 35]. I use *A*_NP-NP_ = 30 × 10^− 20^ J (or $\simeq 70$*k*_B_*T* provided *T* = 300 K).

For the SiO_2_-SiO_2_ vdW interaction, I employ the Hamaker constant, *A*_NP-NP_ = 0.2 × 10^− 20^ (or $\simeq 0.5$*k*_B_*T*), which is close to those calculated and measured in Refs. [36] and [37], respectively.

For the vdW interaction between proteins in the native folded state, the Hamaker constants provided by the experiment and theory are in the range from 0.4 to 4 × 10^− 20^ J, i.e., 1-10 *k*_B_*T* [33, 34]. In the corona around NPs, the proteins are expected to be denatured at least partly [7], and this can influence the Hamaker constant. For example, the fit of the experimental data indicates that the corresponding Hamaker constant for HSA is $\simeq ~10$*k*_B_*T* (see the Supporting Information in Ref. [15]). I employ *A*_PC-PC_ = 1.2 × 10^− 20^ J or $\simeq 3$*k*_B_*T* as in Ref. [34]. The increase of this constant up to 10 *k*_B_*T* does not change the main conclusions, because in the context under consideration, the main role of proteins is rather in creation of steric constraints for contacts of NPs than their contribution to the vdW interaction.

For the Au-protein vdW interaction, I have estimated the Hamaker constant, *A*_NP-PC_ = 10 × 10^− 20^ (or $\simeq 25$*k*_B_*T*), by using the Lifshitz theory (as was earlier detailized in Ref. [38]). For the SiO_2_-protein vdW interaction, similar estimates have resulted in *A*_NP-PC_ = 0.5 × 10^− 20^ (or $\simeq 1$*k*_B_*T*).

To describe the hydration energy, I use *B* = 0.03 J/m^2^ and *α* = 3.8 nm^− 1^. The hydration energy calculated with these parameters is expected to be suitable for various systems (see, e.g., Refs. [29, 39–41]).

The size of NPs employed in the experimental studies related to biological applications is usually between 20 and 180 nm. In my calculations, *R*_1_ and *R*_2_ are chosen to be 20, 50, and 80 nm. The internal long-lived “hard” part of PC is usually considered to contain one or two protein layers, and its thickness is believed to be comparable with the size of large proteins (e.g., HSA). Taking this into account, I use *h*_1_ = *h*_2_ = 5 nm.

## Results of calculations

This work is focused on the vdW and hydration interaction between NPs, and the corresponding results are presented at 0.4 ≤ *d* ≤ 10 nm. The interaction at *d* < 0.4 nm is not shown because in this limit it can be appreciably influenced by the double-layer electrostatic counterpart. The interaction at *d* > 10 nm is relatively weak and accordingly not important.

**Fig. 2:**
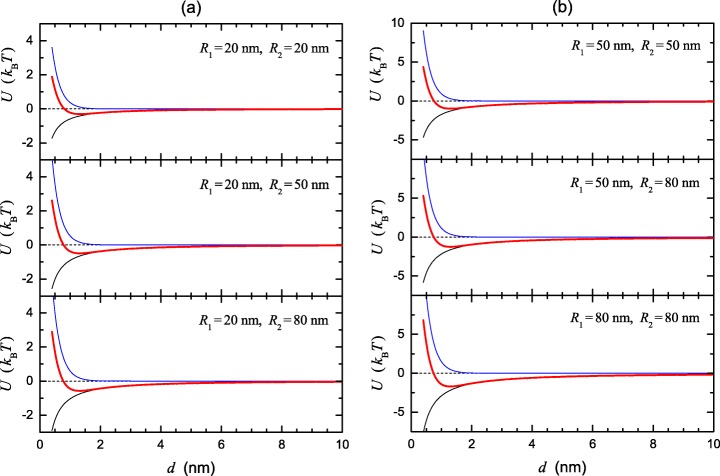
Energy of the interaction between bare SiO_2_ nanoparticles as a function of the minimal distance between them. The van der Waals and hydration interactions are shown by thin solid lines. The whole interaction is represented by a thick solid line

**Fig. 3:**
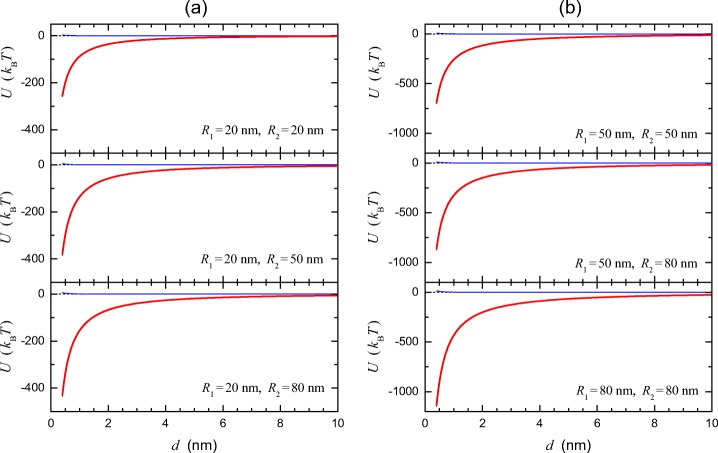
As in Fig. 2 for bare Au nanoparticles

The interaction of bare SiO_2_ NPs is predicted to be fairly weak, its absolute value is typically below 3 *k*_B_*T* (Fig. 2), and accordingly it is not sufficient for aggregation. In contrast, the interaction of bare Au NPs is strong, down to about − 200*k*_B_*T* even in the case of small NPs with *R*_1_ = *R*_2_ = 20 nm (Fig. 3), and it can result in irreversible aggregation.

**Fig. 4:**
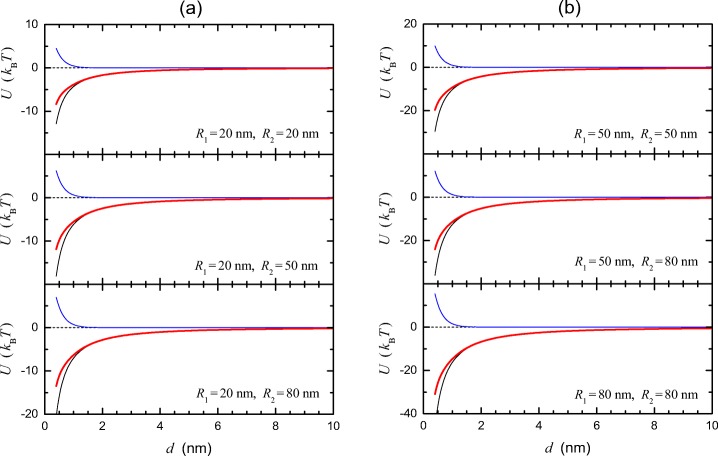
As in Fig. 2 for SiO_2_ nanoparticles with a protein corona

With PC, the interaction of SiO_2_ NPs becomes more appreciable (Fig. 4). If one of the NPs is small (*R*_1_ = 20 nm; Fig. 4a), its absolute value is typically below or comparable with 10 *k*_B_*T* (Fig. 2), and accordingly it is not sufficient for aggregation either. For larger NPs with *R*_1_ = 50 or 80 nm, Fig. 4a), its absolute value can reach 50–70 *k*_B_*T*, and it may be sufficient for aggregation.

**Fig. 5:**
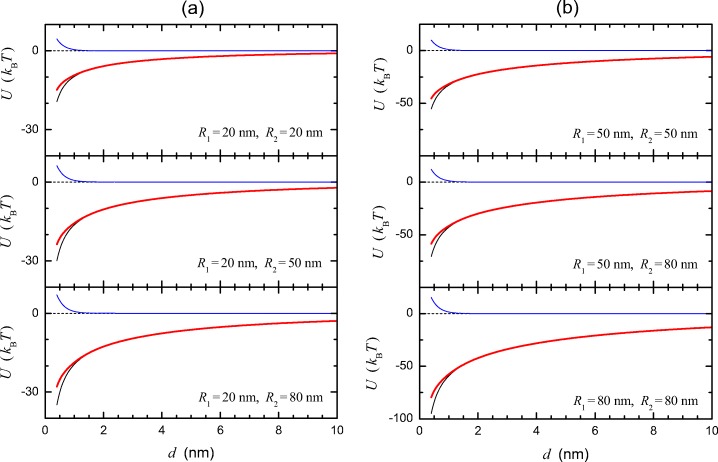
As in Fig. 2 for Au nanoparticles with a protein corona

The formation of PC around Au NPs reduces the interaction between them so that it may be insufficient for aggregation of small NPs (Fig. 5a) but still sufficient for aggregation of large NPs (Fig. 5b).

Comparing the vdW and hydration contribution with the interaction (Figs. 2–5), one notice that the vdW part dominates nearly always.

**Fig. 6:**
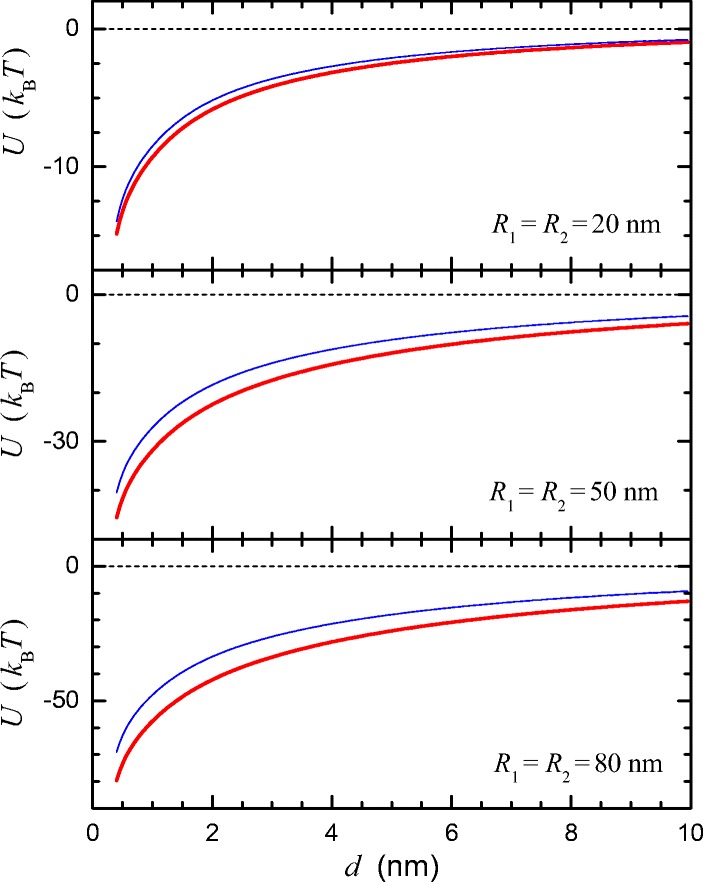
Energy of the interaction between Au nanoparticles with a protein corona as a function of the minimal distance between them. The thick and thin solid lines correspond to *A*_NP-NP_ = 30 × 10^− 20^ J (as in Fig. 5) and 17 × 10^− 20^ J, respectively

As already mentioned, the accuracy of calculated and measured Hamaker constants is not high. Its scale is roughly ± 40*%*. For SiO_2_, with variation of the Hamaker constants in this range, the interaction either remains weak (in the case of bare NPs) or changes only a little (in the case of NPs with PC), i.e., it does not change the conclusions. For Au, the vdW is appreciable, and it is instructive to show explicitly how the results change with, e.g., decreasing *A*_NP-NP_ from 30 × 10^− 20^ J (as has been used in the analysis presented above) to 17 × 10^− 20^ J (as is reported in the most recent calculations [35]). In the case of bare Au NPs (Fig. 3), the vdW interaction fully dominates, and its decrease by 43*%* (from 30 to 17 × 10^− 20^ J) results in the corresponding decrease of the whole interaction. In the case of Au NPs with PC, the contribution of the Au-Au vdW interaction to the whole interaction is smaller, and the reduction of *A*_NP-NP_ by 43*%* results in modest changes of the whole interaction (Fig. 6).

## Conclusion

Taken together, the results of calculations shown in Figs. 2–6 clarify the role of PC in the interaction between NPs. Basically, the analysis presented indicates that in the case of SiO_2_ NPs, the aggregation is usually not important. In contrast, the vdW interaction can easily induce aggregation of bare Au NPs, while the presence of PC can prevent this process at least between small NPs with size up to about 20 nm. Thus, small Au NPs are preferable if the aggregation is undesirable. Large Au NPs are accordingly preferable if the aggregation is useful as it may be, e.g., in hyperthermia therapy. In the case of a broad distribution of NP size (e.g., from 20 to 100 nm), one can expect that small NPs will first rapidly aggregate with large NPs and then this process will be followed by relatively slow aggregation of large NPs.

Finally, note that the results presented can be combined with already available kinetic models of aggregation of NPs [8–12]. In such temporal models, aggregation is usually considered to occur in a fixed volume so that one can operate with the average concentrations of aggregates. Under in vivo conditions, aggregation occurs after injection of NPs, so that the region of the NP location expands due to their diffusion, and accordingly the corresponding models should be spatio-temporal. The analysis of the kinetics belonging to the latter class is of interest both from the points of view of statistical physics and NP applications.
